# Molecular Profiling of Germline Variants in the DNA Mismatch Repair Genes in Chinese Colorectal Cancer Patients

**DOI:** 10.1155/genr/9910339

**Published:** 2026-03-17

**Authors:** Xiu Zhu, Da Han, Jun Chen, Jingjing Xu, Jiaoyue Jin, Qian Lai, Lina Xie, Jianfei Fang, Liu Zhu, Ying Yu, Jun Yang, Taoli Wang, Xueping Xiang, Xuejiang Shi, Desheng Xiao, Dan Su

**Affiliations:** ^1^ Zhejiang Cancer Hospital, Hangzhou Institute of Medicine (HIM), Chinese Academy of Sciences, Hangzhou, China, cas.cn; ^2^ Department of Research and Development, Beijing SinoMDgene Technology Co., Ltd, Beijing, China; ^3^ Department of Pathology, Nanjing Drum Tower Hospital, The Affiliated Hospital of Nanjing University Medical School, Nanjing, China, nju.edu.cn; ^4^ Department of Pathology, Zhuzhou Central Hospital, Zhuzhou, Hunan, China, zzszxyy.cn; ^5^ Department of Pathology, The Second Affiliated Hospital Zhejiang University School of Medicine, Hangzhou, China, z2hospital.com; ^6^ Department of Pathology, Xiangya Hospital Central South University, Changsha, Hunan, China, csu.edu.cn

**Keywords:** colorectal cancer (CRC), germline variant, Lynch syndrome, MMR

## Abstract

**Background:**

A multicenter study on the DNA mismatch repair (MMR) genes enabled us to study the profiling of germline variants in MMR genes of colorectal cancer (CRC) patients with MMR deficiency (dMMR). The clinicopathological differences between Lynch syndrome (LS) patients and sporadic dMMR CRC patients were compared by Student’s *t*‐test and *χ* ^2^ test. The molecular profiling of germline variants in MMR genes in Chinese CRC patients with dMMR is clarified.

**Methods:**

A total of 326 CRC patients with dMMR were enrolled. Next‐generation sequencing (NGS) and Sanger sequencing were performed using tumor‐adjacent tissues of enrolled patients. Four MMR genes (*MLH1*, *MSH2*, *MSH6*, and *PMS2*) are included in the NGS panel.

**Results:**

A total of 113 germline variants were detected, including 81 pathogenic and likely pathogenic variants. The clinicopathologic differences between CRC patients with/without LS were observed in age, family history, lesion location, and dMMR patterns. The CRC cohort with IHC‐MSH6 negative alone shows the highest prevalence rate of LS. *MLH1* was detected with the most germline variants. The mutational hotspot region of *MLH1* is Exon 8, Exon 4 for *MSH6*, Exon 11 for *PMS2*, and Exon 7 for *MSH2*. Several germline hotspots were labeled on each MMR gene sequence by fixed‐size bin analysis. In addition, some variants were novel discovered based on the presence or absence of the RS number and allele frequency record.

**Conclusions:**

Our study classified the clinicopathological features between sporadic CRC patients and LS patients. More importantly, the molecular profiling of the MMR gene germline variant was experimentally elucidated, which deepens the knowledge of MMR genes and provides a new perspective for the subsequent studies on the landscape of germline variants of Chinese LS patients.

## 1. Introduction

Lynch syndrome (LS) is one of the most common familial hereditary cancer predisposition diseases, which has been proven to be associated with increased risk of colorectal cancer (CRC) and other extracolonic tumors [1, 2]. LS is caused by germline pathogenic variants in DNA mismatch repair (MMR) genes, including *MLH1*, *MSH2*, *MSH6*, and *PMS2* [3].

The role of MMR genes is to repair the occasional mismatch variant during DNA replication and maintain the homeostasis of cell function. Some LS cases are due to *MSH2* promoter hypermethylation caused by *EPCAM* deletions [4]. The loss of MMR protein will leave the mismatched genomic DNA unrepaired, resulting in sequence insertion or deletion errors in the microsatellite region of the genome, that is, microsatellite instability (MSI). Germline variants in MMR genes could lead to the loss of DNA MMR function, which consequently results in the pathogenic accumulation of errors during DNA replication, eventually causing tumorigenesis. Germline variant detection of the MMR gene in CRC patients by next‐generation sequencing (NGS) is a necessary method to diagnose LS. In addition, detection of MMR protein expression level by immunohistochemistry (IHC) staining or MSI detection, combined with the patient’s family history, is also commonly used for initial screening of LS.

Several studies reported the gene mutational characteristic of CRC patients. Whole‐genome sequencing of 97 CRC patients showed that 16% were found to be hypermutated, of which three‐quarters had high microsatellite instability (MSI‐H), typically hypermethylation and *MLH1* silencing, and one‐quarter had somatic alterations in the MMR gene and polymerase *ε* (*POLE*) [5]. A study performed germline testing for variants in 25 genes associated with inherited cancer risk for 1,058 North American CRC participants and reported that 105 patients carried one or more pathogenic variants, including 33 (3.1%) with LS [6]. This study suggested that LS probably accounts for 3.1% of CRC patients in the North American population. In the Mexican population, the clinical and molecular spectrum of variants in 412 suspected LS has been reported, and 27.1% (112/414) had a variant in MMR genes, and *MLH1* carried most of the variants [7]. Additionally, a survey in Latin American LS individuals showed the spectrum of pathogenic MMR variants, including the clarification of frequent mutational regions of MMR genes, such as Exon 4 of *MSH6*, which carried the most variants (65%) [8]. The spectrum of MMR variants in the Swedish LS population has been reported, demonstrating that the frequency of *MLH1* variants was the highest (40%), followed by *MSH2* (36%), *MSH6* (18%), and *PMS2* (6%) [9]. In the Chinese population, a study reported that 95 out of 311 MMR deficiency (dMMR) CRC patients carried pathologic germline variants in MMR genes, and among the 95 individuals, approximately 51.6%, 28.4%, 14.7%, and 5.3% of cases carried pathogenic or likely pathogenic germline variants in *MLH1*, *MSH2*, *MSH6*, and *PMS2*, respectively [10]. Another study involving 406 dMMR and 250 MMR‐proficient (pMMR) Chinese CRC individuals reported that 88/154 CRCs were identified as LS and 21 previously unreported pathogenic variants were discovered. They further compared clinical characteristics and genetic features between Chinese LS and sporadic CRC, which concluded the difference in variant frequencies of *APC*, *CREBBP*, and *KRAS*, as well as different upregulation of tumor‐associated signal transduction pathways [11].

At present, the characteristics of the MMR gene germline variant in East Asian CRC populations remain largely unknown. It has been reported that the prevalence of MMR germline variants is low among Japanese cancer patients, with only 3 cases (0.3%) of MMR germline variants among 1058 pan‐carcinoma patients enrolled in this study [12]. In order to characterize the MMR gene germline variants in East Asian tumor populations, a study of an enriched suspected LS cohort is required. In our study, 326 Chinese dMMR CRCs were enrolled, and underwent NGS targeting 4 MMR genes and Sanger sequencing were performed for germline variant reconfirmation. Based on these studies, we systematically described the clinicopathological characteristics and the molecular mutational characteristics of the MMR gene germline variant of Chinese CRC patients.

## 2. Materials and Methods

### 2.1. Ethics Statement

This study was approved by the Medical Ethics Committee of Zhejiang Cancer Hospital (approval no. IRB‐[2022]274), the Medical Ethics Committees of Nanjing Drum Tower Hospital, the Affiliated Hospital of Nanjing University Medical School (approval no. AF/SC‐08/03.0), the Human Research Ethics Committee of the Second Affiliated Hospital Zhejiang University School of Medicine (approval no. IR2022161), and the Medical Ethics Committee of Xiangya Hospital Central South University (approval no. 202206159), and was performed in compliance with the Declaration of Helsinki.

### 2.2. Patients

Formalin‐fixed paraffin‐embedded (FFPE) tumor‐adjacent tissues from a total of 326 CRC patients were collected in the hospitals described above between June 2019 and May 2023. The clinical information of the enrolled patients was collected.

### 2.3. IHC

The IHC was performed using the BenchMark ULTRA Advanced Staining System (Ventana, Tucson, AZ, USA). The experiment was conducted according to the user’s manual. Reagents were loaded into the instrument and recognized by the system. Subsequent steps of instructions, automated dewaxing, hydration, cell permeabilization, antigen repair, serum blocking, antibody incubation, and DAB treatment were performed on the system automatically. Quality control was set up for each run of experiments, which typically includes 1 negative control and 4 positive controls for MLH1, MSH2, MSH6, and PMS2 proteins, respectively. The slides of 1 negative control and 4 MMR proteins were all included. The result with staining of all 4 MMR proteins was determined to be pMMR, and a result with at least 1 negative staining was determined to be dMMR. The antibodies used in this study were purchased from Ventana (Tucson, AZ, USA).

### 2.4. DNA Extraction

No less than 5 FFPE slices (10 μm per slice) were used to ensure sufficient extracted DNA (> 100 ng) for subsequent sequencing. FFPE DNA extraction kits (TIANGEN Biochemical Technology Co., Ltd., Beijing, PRC) were used in this study. All operations were performed following the manufacturer’s protocols. Typically, the following steps of the experiment, dewaxing of the FFPE sample, proteinase K treatment, DNA precipitation, adsorption, rinsing, and elution, were included.

### 2.5. NGS

MMR‐targeted NGS was performed for all 326 enrolled samples. Genomic targeting panel RiskCare 58 (Singlera Genomics Co., Ltd., Shanghai, PRC), including capturing of 4 MMR genes (*MLH1*, *MSH2*, *MSH6*, and *PMS2*), was applied. NGS was performed on NextSeq 500AR (Illumina, CA, USA). The sequencing depth of the target area is not less than 1000×. Most samples were sequenced to a depth of > 2000×. Variant annotations were carried out using Variant Effector Predictor (VEP, Version 84) and ClinVar (Version 202108), and GRCh37 (hg19) as the reference genome. The NCBI reference transcript sequences of *MLH1*, *MSH2*, *MSH6*, and *PMS2* are NM_000249.4, NM_000251.3, NM_000179.3, and NM_000535.7, respectively. Reference transcripts are selected based on the Matched Annotation from the NCBI and EMBL‐EBI (MANE) database recommended by the American College of Medical Genetics and Genomics (ACMG). The experiment operation, data quality control, and data analysis were performed in accordance with the standard operating procedures of Singlera Genomics Co., Ltd. (Shanghai, PRC). The classification of the clinical significance of the variants is according to the criteria of ACMG [13].

### 2.6. Sanger Sequencing

Sanger sequencing was performed to identify germline variants from the detected MMR variants. The specific primers for individual variants were designed by using SnapGene (Version 6.0.2). The annotation of the variants and the design of the primers are based on the *MLH1* reference sequence (NG_007109.2), the *MSH2* reference sequence (NG_007110.2), the *MSH6* reference sequence (NG_007111.1), and the *PMS2* reference sequence (NG_008466.1), respectively. The PCR products were isolated by electrophoresis and purified by using the QIAquick PCR & Gel Cleanup Kit purchased from QIAGEN (Germantown, MD, USA). The ABI 3730XL genetic analyzer was used for sequencing (Applied Biosystems, CA, USA).

### 2.7. Statistical Analysis

IBM SPSS Statistics 21 was used for statistical analysis. Statistical significance was defined by two‐tailed *p* values as ^∗^ *p* < 0.05, ^∗∗^ *p* < 0.01, ^∗∗∗^ *p* < 0.001. *p* values of continuous variables were obtained from *t-*test, and categorical variables from *χ* ^2^ test.

## 3. Results

### 3.1. Overview of Targeted Sequencing of MMR Genes

A total of 326 cases of dMMR CRC patients were enrolled, and targeted sequencing of MMR genes as well as 53 other solid tumor‐associated genes was performed using NGS. A total of 104 patients were detected with germline variants, of which 94 cases (90.4%) carried only one variant, 10 cases (9.6%) were detected with 2 or 3 variants, and 81 cases (77.9%) carried with pathogenic/likely pathogenic germline variants. The pathogenic germline variants may lead to recurrence of CRC or new onset of other LS‐associated tumors, and that family members of patients with LS are at risk for tumor susceptibility. The variant distribution in enrolled patients is detailed in Figure 1(a) and Supporting Table 1. A total of 162 variants were detected in this study, including 113 germline variants. The landscape of the number of germline sporadic variants (benign and likely benign variants are excluded) in MMR genes is detailed in Figure 1(b), which shows the number of germline variants detected in the *MLH1* gene is the largest, accounting for 50 (44.2%), followed by 27 (23.9%) germline variants in *MSH2*, 26 (23%) in *MSH6*, and 10 (8.8%) germline variants in *PMS2* subsequently. Furthermore, 28 cases (24.8%) of the *MLH1* germline variant were pathogenic, and 12 cases (10.6%) were likely pathogenic, representing the most LS‐associated variants detected among all MMR genes. The clinical consequence of detected germline variants in other MMR genes is shown in Figure 1(c).

FIGURE 1Overview of MMR gene variants detected in this study. (a) Summary of carriers of germline variants in enrolled patients. (b) Summary of variants detected in each MMR gene. (c) Summary of clinical significance of detected germline variants on each MMR gene. Abbreviations: PV, pathogenic variants; VLP, variants of likely pathogenic; VUS, variants of uncertain significance.

**Figure:**
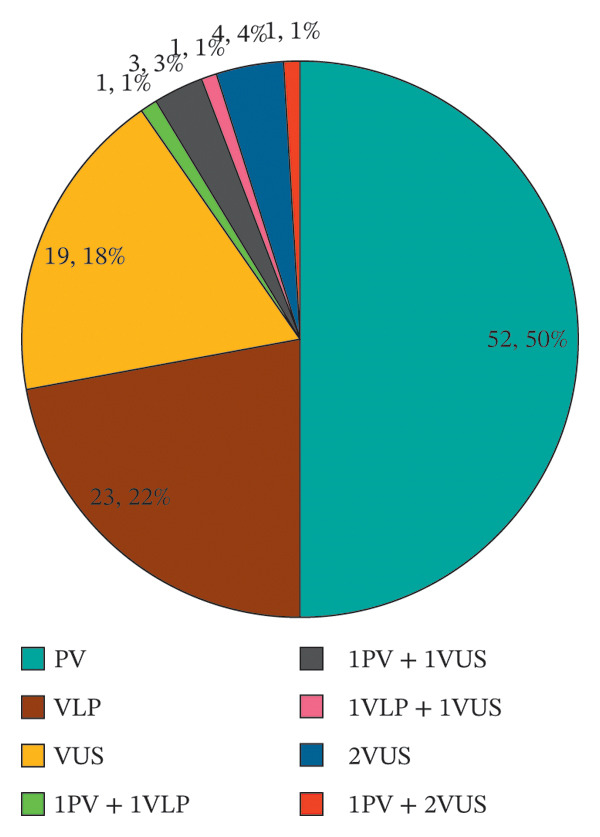
(a)

**Figure:**
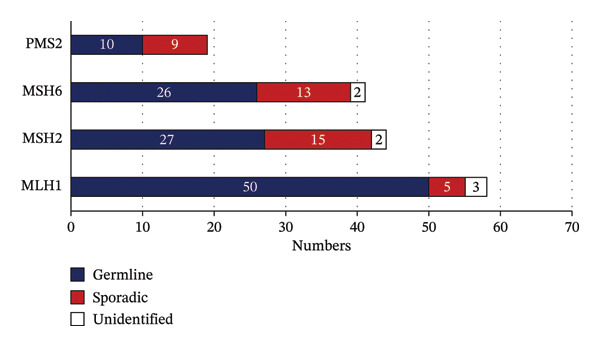
(b)

**Figure:**
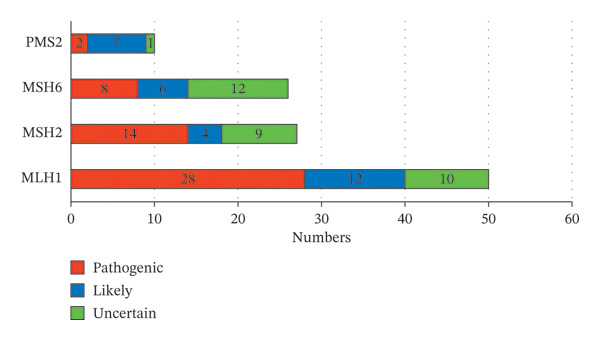
(c)

### 3.2. Clinicopathological Characteristic

To identify the clinical characteristics of LS patients in the Chinese CRC population, 326 dMMR CRC patients were divided into two groups, CRC patients with LS (81 cases, hereafter referred to as the LS group) and dMMR CRC patients without LS (245 cases, hereafter referred to as the non‐LS group), according to whether germline pathogenic or likely pathogenic variant was detected. The comparison of clinicopathological characteristics of the LS group and the non‐LS group is shown in Table 1. Firstly, the mean age of LS patients is 50.9 years, which is significantly younger than the non‐LS group (*p* < 0.001). Secondly, when comparing the above two groups, a low significant difference in the distribution of tumor lesions is observed (*p* = 0.008). Thirdly, the percentage of LS patients with recorded family history was significantly higher than that of the non‐LS group (*p* < 0.001), which is consistent with common sense. Lastly, the patterns of MMR gene expression loss, presented by IHC, show low significant difference between the LS and non‐LS groups (*p* = 0.013).

**TABLE 1:** Clinical features of dMMR CRC patients with LS (LS group) and without LS (non‐LS group).

Characteristics	Number of cases	LS (*n* = 81)	Non‐LS (*n* = 245)	*p* ^†^
Age (mean ± SD)	326	50.9 ± 12.9	60.1 ± 11.0	*p* < 0.001
Sex				*p* = 0.268
Female	142	31 (38.3%)	111 (45.3%)	
Male	184	50 (61.7%)	134 (54.7%)	
Location				*p* = 0.008
Right colon	168	34 (42.0%)	134 (54.7%)	
Left colon	64	20 (24.7%)	44 (18.0%)	
Colon	16	7 (8.6%)	9 (3.7%)	
Transverse colon	19	6 (7.4%)	13 (5.3%)	
Rectum	50	8 (9.9%)	42 (17.1%)	
Multiple lesions	9	6 (7.4%)	3 (1.2%)	
Family history				*p* < 0.001
No family history	281	61 (75.3%)	220 (89.8%)	
CRC‐related family history	27	16 (19.8%)	11 (4.5%)	
Other cancers	18	4 (4.9%)	14 (5.7%)	
IHC				*p* = 0.013
MSH6 negative alone	31	13 (16.0%)	18 (7.3%)	
PMS2 negative alone	64	16 (19.8%)	48 (19.6%)	
MSH2 negative alone or both MSH2, MSH6 negative	68	19 (23.5%)	49 (20.0%)	
MLH1 negative alone or both MLH1, PMS2 negative	146	31 (38.3%)	115 (46.9%)	
MSH6, MLH1, MSH2, PMS2 negative	2	1 (1.2%)	1 (0.4%)	
Others	15	1 (1.2%)	14 (5.7%)	

^†^ *p* values of continuous variables obtained from *t*‐test and categoric variables from *χ* ^2^ test.

The LS prevalence in the population with different MMR‐deficiency patterns is further checked. The detection of LS‐associated germline variants of MMR genes in populations with various MMR‐deficiency patterns is shown in Table 2. In this study, LS‐associated germline variants are detected in patients with 6 types of MMR patterns and 4 groups are involved for further analysis as their sample size is sufficient. Conclusively, a pathogenic or likely pathogenic variant is detected in 13 (41.9%) patients among the 31 cases (8.2%) of the MSH6‐negative‐alone group, in which LS has the highest prevalence. The other groups were listed in descending order of LS prevalence as follows: MSH2 negative alone or both MSH2 loss and MSH6 loss (19 cases, 27.9%), PMS2 deficiency alone (16 cases, 25.0%), and MLH1 deficiency alone or both MLH1 and PMS2 deficiency (31 cases, 21.2%).

**TABLE 2:** Prevalence of LS in CRC patients with different MMR‐deficiency patterns.

IHC	Number of cases (%)^a^	Number of LS cases (%)^b^		MSH2	PMS2	Total	Number of MSS/MSI‐L (%)^b^
		MSH6	MLH1				
MLH1 negative alone or both MLH1, PMS2 negative	146 (44.8)	1 (0.7)	30 (20.5)	/	/	31 (21.2)	8 (5.5)
MSH2 negative alone or both MSH2, MSH6 negative	68 (20.9)	/	1 (1.5)	16 (23.5)	2 (2.9)	19 (27.9)	3 (4.4)
PMS2 negative alone	64 (19.6)	/	8 (12.5)	1 (1.6)	7 (10.9)	16 (25.0)	8 (12.5)
MSH6 negative alone	31 (9.5)	12 (38.7)	/	1 (3.2)	/	13 (41.9)	7 (22.6)

^a^Percentage = N/326 cases of dMMR CRC.

^b^Percentage = N/cases of corresponding dMMR patterns.

### 3.3. The Molecular Profiling of the MMR Gene Germline Variant of Enrolled CRC Patients With LS

To elucidate the genomic profile of LS in CRC patients, the genomic location of each detected variant was summarized and the mutational hotspots in the whole region of each MMR gene were labeled.

Firstly, the germline variants detected on each MMR gene were summarized and classified by clinical significance, variant type, and consequence. As mentioned above, among the 113 detected germline variants, 50 (44.2%), 27 (23.9%), 26 (23%), and 10 (8.8%) are detected in *MLH1*, *MSH2*, *MSH6*, and *PMS2*, respectively (Figure 1(b)). Of all the 81 cases of pathogenic and likely pathogenic germline variants, the variants on *MLH1* counted up to 40 cases (49.4%), and those on *MSH2*, *MSH6,* and *PMS2* followed with 18 cases (22.2%), 14 cases (17.3%), and 9 cases (11.1%), respectively (Figure 1(c)). Additionally, the proportions of types and consequences of variants in MMR genes detected by NGS are shown in Supporting Figure 1. Among all variants of clinical significance detected in this study, the number of SNPs detected on 4 MMR genes was more than half, whereas in the pathogenic variants associated with LS, INDEL accounted for an increased proportion (Supporting Figure 1A). Similarly, among pathogenic variants, the proportion of missense variants decreased, and that of nonsense and frameshift increased compared with the proportion in variants with all clinical significance (Supporting Figure 1B).

Next, the distribution of all detected germline variants on each exon/intron of the MMR genes is summarized. In Figure 2, variants on the exons of each MMR gene were classified and counted according to clinical significance. In *MLH1*, Exon 8 carries the biggest number of germline variants (9 cases, 18%), followed by Exon 12 and Exon 16 (4 cases, 8%, respectively), and the number of pathogenic variants detected in Exon 16 is the highest (4 cases, 8%) (Figure 2(a)). In *MSH6*, the number of germline variants detected on Exon 4 and Exon 5 is 21 cases, accounting for 80.8% of all germline variants detected on *MSH6* (Figure 2(b)). Among *PMS2*, Exon 11 has the most variants (5 cases, 50%) (Figure 2(c)). Among *MSH2*, Exon 7 has the largest number of variants (5 cases, 18.5%), while Exon 11 and Exon 12 have the largest number of pathogenic variants (3 cases, 11.1%, respectively) (Figure 2(d)).

FIGURE 2Summary of germline variants on exons/introns of each MMR gene.

**Figure:**
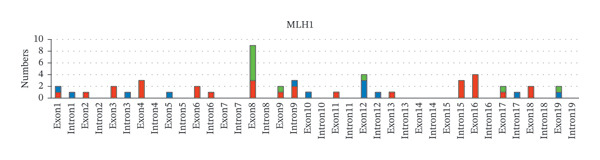
(a)

**Figure:**
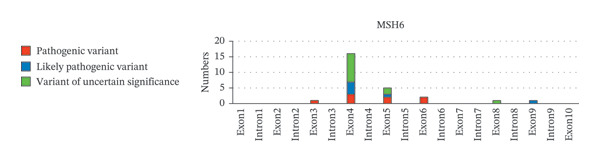
(b)

**Figure:**
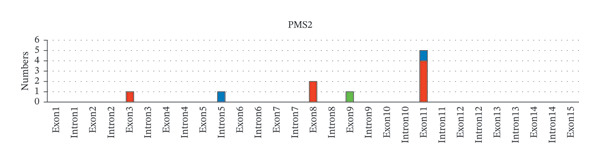
(c)

**Figure:**
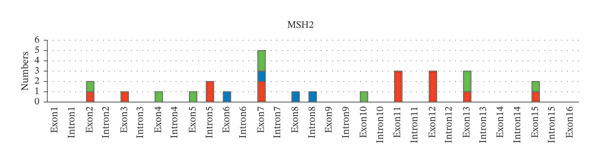
(d)

Furthermore, we tried to figure out certain germline variant hotspots on the MMR genes; thus, the mutational hotspots were summarized by counting the number of variants in each 50‐bp interval of the transcript sequence of each MMR gene. 240 germline variants were used for mutational hotspot analysis, from our study, the study of Yang, M. *et al.* [10], and the study of Li, Y. *et al.* [11], respectively. The detailed distribution of variants in the intervals of each MMR gene is shown in Figure 3. In *MLH1*, the germline variants are distributed in 34 of 50 intervals, and 24 intervals have more than 1 variant (Supporting Table 2). Specifically, interval 1851–1900 bp (in Exon 16), interval 201–250 (across 2 and Exon 3), interval 651–700 bp (in Exon 8), and interval 701–750 bp (across Exon 8 and Exon 9) have the biggest number of variants (29/93, 31.2%), suggesting that these regions are potential mutational hotspots in *MLH1*. The number of variants in other intervals was detailed in Figure 3(a). Similarly, the reference sequences of *MSH2*, *MSH6*, and *PMS2* were divided into 63, 86, and 102 intervals (50 bp each), and the germline variants are distributed in 30, 36, and 8 of those, respectively. In *MSH2*, interval 1701–1750 bp (in Exon 11), interval 1251–1300 bp (in Exon 7), and interval 1201–1250 bp (in Exon 7) have the biggest number of variants (29/77, 37.7%) (Figure 3(b)). In *MSH6*, interval 3301–3350 bp (in Exon 5), interval 3551–3600 bp (in Exon 6), interval (3601–3650 bp (across Exon 6 and Exon 7), and interval 3251–3300 (across Exon 4 and Exon 5) have the biggest number of variants (19/54, 35.2%) (Figure 3(c)). In *PMS2*, interval 1751–1800 bp (in Exon 11) has the biggest number of variants (7/16, 43.8%). The intervals with 2 or more variants of each MMR gene were listed in Supporting Table 2. These studies demonstrated the existence of germline variant hotspots on the MMR genes.

FIGURE 3The distribution of germline variants in the reference transcriptional sequences of each MMR gene.

**Figure:**
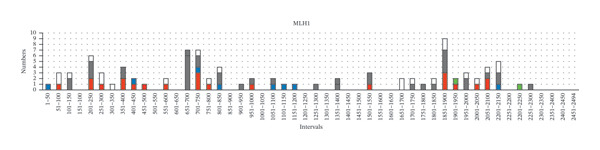
(a)

**Figure:**
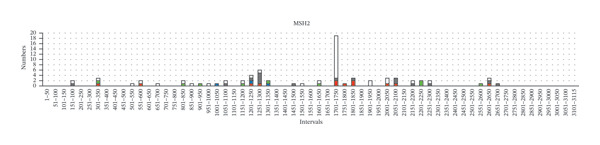
(b)

**Figure:**
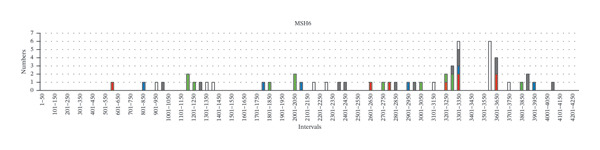
(c)

**Figure:**
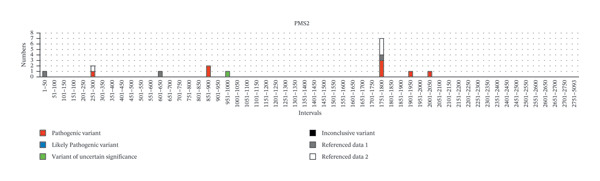
(d)

Additionally, several single nucleotide sites were observed as potential hotspots, as those SNPs detected in this study were observed to be shared by multiple patients. Detailed information on these variants and their allele frequency data in the Asian population is presented in Supporting Table 3. Notably, 7 variants, including *MLH1*: c.1731+1G>A, *MLH1*: c.1852_1854del, *MLH1*: c.676C>T, *PMS2*: c.861_864del, *MSH2*: c.942+3A>T, *MLH1*: c.350C>T, and *MSH6*: c.3261del/dup, are shared by multiple carriers in this study, but the allele frequency data of these variants in Asian populations are unrecorded. Consequently, the recorded allele frequency data in the Asian population for all the detected variants in this study were summarized. The result shows that 125 cases (125/165, 75.8%) are lacking the allele frequency data in the Asian population (Supporting Table 5). Additionally, *MSH2*: c.1699A>T, reported by Li, Y. *et al.* [11], is shared by 16 patients, of which the allele frequency data are absent in the Asian population in the NCBI database (Supporting Table 6).

Meanwhile, several variants are *novel* discoveries. In our study, 19 cases (16.8%) of germline variants lacked a reference SNP number, that is, were without record in the ClinVar database, suggesting these variants are novel discoveries (Table 3). Notably, *MLH1*: c.1852_1854del has been previously reported by Yang, M. *et al.* [10] and Li, Y. *et al.* [11], indicating this variant has a certain prevalence in Chinese CRC patients with LS.

**TABLE 3:** Novel germline variants of MMR genes in Chinese patients with CRC.

Gene	Exon/intron	HGVSc	HGVSp	Clinical significance	Variant type	Variant consequence
MLH1	Exon 1	c.27del	p.Arg10GlyfsTer7	Pathogenic	INDEL	Frameshift
MLH1	Exon 3	c.210_213del	p.Glu71IlefsTer20	Pathogenic	INDEL	Frameshift
MLH1	Exon 3	c.240dup	p.Thr81HisfsTer3	Pathogenic	INDEL	Frameshift
MLH1	Exon 6	c.463dup	p.Leu155ProfsTer17	Pathogenic	INDEL	Frameshift
MLH1	Exon 10	c.811del	p.Ser271ProfsTer2	Likely	INDEL	Frameshift
MLH1	Exon 11	c.929del	p.Thr310LysfsTer57	Pathogenic	INDEL	Frameshift
MLH1	Exon 12	c.1065del	p.Ser356LeufsTer11	Likely	INDEL	Frameshift
MLH1	Exon 12	c.1080del	p.Lys361AsnfsTer6	Likely	INDEL	Frameshift
MLH1	Exon 12	c.1168del	p.Glu390AsnfsTer11	Likely	INDEL	Frameshift
MLH1	Exon 16	c.1852_1854del	p.Lys618del	Pathogenic	INDEL	In‐frame deletion
MSH2	Exon 7	c.1165del	p.Arg389Aspfs^∗^23	Likely	INDEL	Frameshift
MSH2	Exon 8	c.1281del	p.Lys427AsnfsTer11	Likely	INDEL	Frameshift
MSH2	Exon 11	c.1720dup	p.Gln574ProfsTer6	Pathogenic	INDEL	Frameshift
MSH6	Exon 4	c.2002del	p.Ser668Profs^∗^4	Likely	INDEL	Frameshift
MSH6	Exon 4	c.2549dup	p.Tyr850^∗^fs^∗^1	Pathogenic	INDEL	Frameshift
MSH6	Exon 4	c.2850_2868del	p.Ser950ArgfsTer12	Likely	INDEL	Frameshift
MSH6	Exon 4	c.3141_3144dup	p.Ser1049AlafsTer18	Pathogenic	INDEL	Frameshift
MSH6	Exon 4	c.753dup	p.Ser252IlefsTer4	Likely	INDEL	Frameshift
PMS2	Exon 11	c.1997_1998del	p.Lys666Argfs^∗^4	Likely	INDEL	Frameshift

*Note:* The “significance” (referring to clinical significance in the database) was queried from the NCBI ClinVar database (URL: https://www.ncbi.nlm.nih.gov/clinvar). Since the data were not obtained through experiments, analysis of significant differences cannot be performed.

## 4. Discussion

In this study, 326 dMMR CRC patients were enrolled, and NGS analysis was performed. The targeted panel covers 4 MMR genes. Sanger sequencing was performed to verify whether the detected MMR variants were germline. According to NGS and germline reconformation data of Sanger sequencing, the patients enrolled in this study were divided into two categories: CRC patients with LS and dMMR CRC patients without LS. The clinicopathologic features of the two groups of dMMR patients were compared. The differences observed in this study were mainly caused by age, tumor lesion location, and pattern of dMMR. The prevalence of LS was highest in patients with IHC‐MSH6 deficiency alone. More importantly, the potential variant hotspots on each MMR gene were identified by labeling the genomic distribution of detected germline variants. In addition, the allele frequency data for all the detected MMR gene variants were searched in the NCBI database, and no relevant records for some variants in Asian populations have been made so far. Notably, several novel discovered germline variants are detected in the present study. In summary, at the first attempt, our study performed the systematical analysis of characterizing the molecular signatures of MMR germline variants in Chinese dMMR CRC patients with LS. A patient carrying a pathogenic germline variant of the MMR gene is diagnosed to have LS. LS patients and their family members with variants have a significantly higher risk of developing LS‐related tumors compared to the general population; thus, performing regular colonoscopies or other management strategies is important for people with LS.

This study distinguished LS patients from non‐Lynch individuals solely by SNV/INDEL in MMR genes and further analyzed hotspots of germline variants in MMR genes. In fact, the absence of data of structural variants in MMR genes/*EPCAM* has limitations, which may lead to missed diagnosis of some LS patients. Among LS patients, germline structural variants in MMR genes and *EPCAM* account for a certain proportion. In Chinese LS patients, the proportion of those carrying large deletions/duplications in MMR genes is approximately 15%–21%, while the proportion of germline variations in the *EPCAM* in the Chinese population is about 3% [6, 14–17]. Large genomic deletions/duplications in MMR genes hinder the expression and function of MMR proteins, rendering DNA mismatches unable to be effectively repaired, thereby significantly increasing the risk of cancer [17]. For example, *MLH1* rearrangement results in MLH1 protein loss, ultimately resulting in dMMR. Germline deletions in the *EPCAM* lead to epigenetic silencing of the downstream *MSH2*, preventing the normal expression of the MSH2 protein, disrupting the MMR system, and eventually leading to the development of LS [18]. Genomically, *EPCAM* is adjacent to *MSH2* on Chromosome 2; thus, structural variants in the *EPCAM* lead to EPCAM‐MSH2 fusion, which subsequently silences MSH2 protein expression [19]. Since the absence of large genomic rearrangement detection, part of LS is potentially omitted among the enrolled dMMR patients in this study. The limitations of routine molecular testing employed in this study are unavoidable; however, they exert a limited impact on the core conclusion, which is to establish the germline variant hotspots of MMR genes.

It is worth noting that the underlying mechanisms of the inconsistent cases between IHC and NGS results in this article need to be clarified. In MMR gene mutation detection, inconsistencies between NGS and IHC results may be driven by multiple mechanisms. Particularly, in the cases identified in this study—where IHC showed loss of MLH1/PMS2 but the diagnosis was MSH6‐associated LS, and where IHC indicated loss of MSH2/MSH6 but pathogenic germline mutations in PMS2 were detected—the core reasons can be attributed to the interplay between technical limitations and biological complexity [20, 21]. For one, from a technical perspective, IHC results are susceptible to antibody specificity bias, tissue fixation quality, and subjectivity in interpretation, which may lead to misjudgment of protein deficiency patterns [22]; however, the limitations of NGS with region‐specific hybridization capture used in this study in detecting large fragment rearrangements, noncoding region mutations, or epigenetic variations may lead to missed detection of key variants. For another, from the analysis of biological mechanisms, the complex dependence of MMR proteins (MLH1 forms functional dimers with PMS2, and MSH2 forms functional dimers with MSH6) makes it possible that a single gene defect may cause co‐deletion of related proteins through imbalance of protein stability. For example, *PMS2* mutations may indirectly lead to MLH1 degradation [23]. In addition, somatic events (such as *MLH1* promoter methylation and somatic mutations in *MSH2*) can cause tumor cells to exhibit a protein deficiency phenotype inconsistent with germline mutations, while germline mosaicism or rare epigenetic mutations further increase the possibility of differences in results [24]. The aforementioned mechanisms suggest that due to differences in methodologies and the complexity of molecular mechanisms, there may be nuances in the diagnosis of dMMR when using different experimental methods. In dealing with certain controversial cases, it is necessary to integrate multiple technical approaches to avoid the inherent limitations of a single method.

About the prevalence rate of LS in cancer patients, there are some similarities and differences between our conclusions and those of other studies. Firstly, our data on the prevalence rate of LS in dMMR CRC are consistent with those of other groups in China. In our study, 81 out of 326 dMMR CRCs (24.8%) are identified as LS, while that of Yang, M. *et al.* is 30.5% (95/311) [10] and that of Li, Y. *et al.* is 21.4% (87/406) [11]. Secondly, we also noticed, with regard to the prevalence rate of LS in the MSI‐H population, that the incidence of LS in CRC is higher than that in the pan‐cancer population enrolled by a group from North America. A study designed to demonstrate that the MSI test/MMR‐IHC could be applied for LS screening in pan‐cancer showed that of 15,045 patients in 50 multiple cancer types, 326 patients were identified as MSI‐H, and 53 (53/326, 16.3%) were diagnosed with LS [25]. In our study, 73 (25.6%) of the 285 CRC patients with MSI‐H were identified as LS, and 53 (18.6%) of them carried pathogenic germline variants in MMR genes (Supporting Table 4), which is higher than that observed in the North American population of pan‐cancer with MSI‐H. Thirdly, the variant rate of MMR genes of Chinese dMMR CRC patients is higher than that in the Mexican cohort of likely LS. In a study of a Mexican group, the clinical and molecular features of 414 likely LS individuals have been reported as 27.1% (112/414) had a variant in MMR genes, and *MLH1* was the most frequently mutated gene [7], whereas 31.9% (104/326) of patients were detected with germline variants, and *MLH1* carried most variants in our study.

About the profiling of germline variants of MMR genes, our study was compared with former studies from mainland China and other countries and regions. Firstly, our study reconfirmed the most frequently mutated MMR gene. Yang, M. *et al.* reported that the number of pathogenic and likely variants in *MLH1*, *MSH2*, *MSH6*, and *PMS2* are 47.3%, 22.6%, 19.4%, and 9.7%, and the prevalence rate of LS was the highest in dMMR CRC patients with IHC‐MSH6 loss‐alone (53.1%) [10], which is consistent with our findings. In a study conducted by a Swedish team, a total of 201 variants of the MMR gene were detected in 369 LS families, of which *MLH*1 variants were detected in 40% of families, *MSH2* in 36%, *MSH6* in 18%, and *PMS2* in 6% [9], which is consistent with our conclusion. Additionally, among the 3000 unique germline sequence variants of MMR genes recorded in the International Society for Gastrointestinal Hereditary Tumors (InSiGHT) database, *MLH1*, *MSH2*, *MSH6,* and *PMS2* account for 40%, 34%, 18%, and 8%, respectively [26, 27], which is consistent with our study. Interestingly, the conclusions of the most frequently mutated exons of each MMR gene are inconsistent between our study and former studies. A research in Latin American LS individuals reported that the most frequent regions of *MLH1* were Exon 11 (15%), followed by Exons 3 and 7 of *MSH2* (17% and 15%, respectively), Exon 4 of *MSH6* (65%), and Exons 11 and 13 of *PMS2* (31% and 23%, respectively) [8]. Our discrepant conclusion is that the number of variants on Exon 11 of *MLH1* is not the majority, instead of Exon 8, and no germline variant was detected on Exon 13 of *PMS2*. More importantly, since the length of the base pair of Exon 4 of *MSH6* is significantly larger than that of other exons, the reference sequence of MMR genes in our study is divided into several intervals with equal length, which is more meaningful for the study of detailed mutational hotspots on the MMR gene. Moreover, compared with previous studies, our study showed similarities and differences in the most common variant type of MMR genes. Among all disease‐predisposing variants in Swedish individuals, the most common type of variant for *MLH1* is the splice site variant, and the most common type for *MSH2* and *MSH6* is the frameshift variant [9]. Differently, in our study, missense and splice site variants are the most common types among pathogenic variants of *MLH1*, and nonsense variants are the most frequent variant for *MSH2*, which is different from the Swedish study. For *MSH6* is a frameshift variant, which is consistent with that of the Swedish population. Additionally, in the InSiGHT database, most *MLH1*, *MSH2*, and *MSH6* variants are truncating (predominantly nonsense or frameshift variants), and the missense variant is significant (30%–60%) for the 4 MMR genes. In our study, most pathogenic variants of *MLH1*, *MSH2*, *MSH6,* and *PMS2* are nonsense or frameshift variants, and missense variants have a significant proportion among the variants of *MLH1*, *MSH2,* and *MSH6*, which is consistent with the data deposited to the InSiGHT database.

Regarding the molecular characteristics of the MMR gene variant in the Chinese cohort, there are some differences in conclusions between our study and those of other groups. The study of Zhang, L. *et al.* concluded that the coding variants detected in MSH6 accounted for the largest proportion of all MMR genes in the Chinese general population [28], whereas the largest number of germline variants were detected on MLH1 (50 cases, 44.2%) in our study. By comparison, our study is based on targeted NGS, and the enrolled cohort in our study is CRC patients, whereas the study of Zhang, L. *et al.* is based on whole genome sequencing (WGS) in the general population. For the CRC population, we are inclined to believe that the data from targeted NGS are more accurate since the sequencing depth is higher. In addition, due to the differences in the method of interval division, the conclusion about the coding variant hotspots of MMR genes is different between this study and that of Zhang, L. *et al.* [28].

In terms of the prognostic significance of novel discoveries in Table 3, we followed up some patients carrying novel discovered pathogenic germline variants. We found that some of them had recurrences of CRC within 5 years, and some of them developed endometrial cancer again within 5 years, which indicates that those variants probably lead to the susceptibility of CRC and other LS‐associated tumors. Since all of the patients enrolled in our trial were diagnosed within 5 years, the prognosis for most of the patients is good. Our further investigations are being performed to classify this issue.

In addition, some germline variants of uncertain significance (VUS) detected in this study may also be potentially associated with the pathogenesis of LS‐associated CRC. There were a large number of germline VUS detected in our research, which accounts for 28.3% (32/113) of all the detected germline variants of MMR genes. Among them, *MLH1*: c.649C>T was observed to be shared by 6 enrolled patients in our study and with certain allele frequencies deposited in multiple population databases. Notably, two patients with single *MLH1*: c.649C>T and single *MSH2*: c.2203A>G, respectively, have a family history of CRC (Supporting Table 2). Since the patients with these two germline VUS have a certain onset and family history of CRC, it is considered that these variants may increase the risk of LS‐associated CRC. Thus, whether the clinical significances of these two VUS should be elevated, further investigations are required.

## Funding

No funding was received for this research.

## Conflicts of Interest

Xuejiang Shi, Da Han, Liu Zhu, and Ying Yu are employed by Beijing SinoMDgene Technology Co., Ltd. The other authors declare no conflicts of interest.

## Supporting Information

Supporting Information File Name: Supporting Information‐20240822. docx.

Description:

Supporting Figure 1: Summary of the types and consequences of detected germline variants.

Supporting table 1: Summary of the number of patients with different numbers of variants. Supporting table 2: Summary of potential variant hotspots on MMR genes.

Supporting table 3: Summary of variants with multiple carriers and corresponding allele frequency data.

File Name: Supporting Information‐20240822. xlsx.

Supporting table 4: Summary of the information of the enrolled patients. Supplementary table 5: The allele frequencies of detected variants. Supplementary table 6: The information of the referenced data from Yang, M. *et al.* [10] and Li, Y. *et al.* [11].

## Supporting information


**Supporting Information** Additional supporting information can be found online in the Supporting Information section.

## Data Availability

The data that support the findings of this study are available from the corresponding author upon reasonable request.
