# Supplementary material for: Molecular Profiling of Germline Variants in the DNA Mismatch Repair Genes in Chinese Colorectal Cancer Patients
Source: Genet Res (Camb). 2026 Mar 17;2026:9910339. doi: 10.1155/genr/9910339 (PMC13140940; doi:10.1155/genr/9910339)
Supplement: Supplementary file 1 — Supporting Information Additional supporting information can be found online in the Supporting Information section. [file GENR-2026-9910339-s001.docx]

**Supplementary figure 1:**

(Summary of the types and consequences of detected germline variations. (A) The proportion of germline variation types of each MMR gene. (B) The proportion of germline variation consequences of each MMR gene. Abbreviations: PV, pathogenic variation; VLP, variants of likely pathogenic; VUS, variants of uncertain significance; SNP, single nucleotide polymorphism; INDEL, insertion-deletion.)


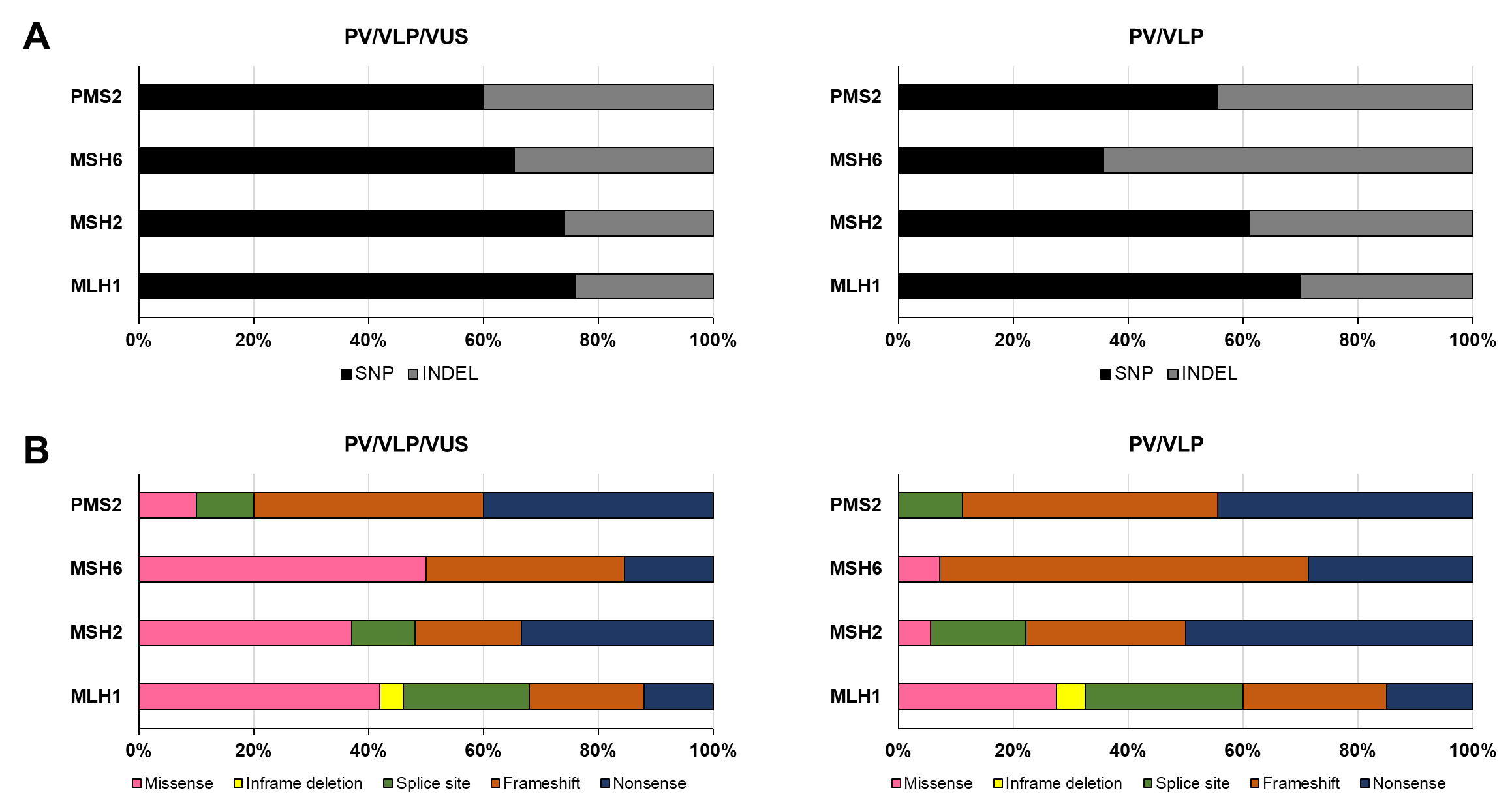


**Supplementary table 1:**

(Summary of the number of patients with different numbers of variants.)

| **Classification** | **Number of cases** | **(n/104) %** |
| --- | --- | --- |
| Patients with germline variants | 104 | / |
| Patients with PV/VLP | 81 | 77.9% |
| with 1 variant | 94 | 90.4% |
| PV | 52 | 50.0% |
| VLP | 23 | 22.1% |
| VUS | 19 | 18.3% |
| with 2 variants | 9 | 8.7% |
| 1PV+1VLP | 1 | 1.0% |
| 1PV+1VUS | 3 | 2.9% |
| 1VLP+1VUS | 1 | 1.0% |
| 2VUS | 4 | 3.8% |
| with 3 variants | 1 | 1.0% |
| 1PV+2VUS | 1 | 1.0% |

**Supplementary table 2:**(Summary of potential variation hotspots on MMR genes.)

| **Gene** | **Intervals (50bp)** | **Genomic location** | **Number of variants** | |  |  |  |  |  |
| --- | --- | --- | --- | --- | --- | --- | --- | --- | --- |
|  |  |  | **Total** | **Data of this study** | |  |  | **Referenced data 1** | **Referenced data 2** |
|  |  |  |  | **Total** | **Pathogenic** | **Likely** | **Uncertain** |  |  |
| MLH1 | 1851-1900 | Exon16 | 9 | 3 | 3 | 0 | 0 | 4 | 2 |
|  | 201-250 | Exon2-Exon3 | 6 | 2 | 2 | 0 | 0 | 3 | 1 |
|  | 651-700 | Exon8 | 7 | 6 | 0 | 0 | 6 | 1 | 0 |
|  | 701-750 | Exon8-Exon9 | 7 | 4 | 3 | 1 | 0 | 2 | 1 |
|  | 2101-2150 | Exon18-Exon19 | 5 | 1 | 0 | 1 | 0 | 2 | 2 |
|  | 351-400 | Exon4 | 4 | 2 | 2 | 0 | 0 | 2 | 0 |
|  | 801-850 | Exon9-Exon10 | 4 | 1 | 0 | 1 | 0 | 2 | 1 |
|  | 2051-2100 | Exon18 | 4 | 2 | 2 | 0 | 0 | 1 | 1 |
|  | 51-100 | Exon1 | 3 | 1 | 1 | 0 | 0 | 0 | 2 |
|  | 101-150 | Exon1-Exon2 | 3 | 0 | 0 | 0 | 0 | 2 | 1 |
|  | 251-300 | Exon3 | 3 | 1 | 1 | 0 | 0 | 0 | 2 |
|  | 1501-1550 | Exon13 | 3 | 1 | 1 | 0 | 0 | 2 | 0 |
|  | 1951-2000 | Exon17 | 3 | 0 | 0 | 0 | 0 | 2 | 1 |
|  | 401-450 | Exon4-Exon5 | 2 | 2 | 1 | 1 | 0 | 0 | 0 |
|  | 551-600 | Exon6-Exon7 | 2 | 1 | 1 | 0 | 0 | 0 | 1 |
|  | 751-800 | Exon9 | 2 | 1 | 1 | 0 | 0 | 0 | 1 |
|  | 951-1000 | Exon11 | 2 | 1 | 1 | 0 | 0 | 1 | 0 |
|  | 1051-1100 | Exon11-Exon12 | 2 | 1 | 0 | 1 | 0 | 1 | 0 |
|  | 1351-1400 | Exon12 | 2 | 1 | 0 | 0 | 1 | 1 | 0 |
|  | 1651-1700 | Exon14-Exon15 | 2 | 0 | 0 | 0 | 0 | 0 | 2 |
|  | 1701-1750 | Exon15 | 2 | 0 | 0 | 0 | 0 | 1 | 1 |
|  | 1801-1850 | Exon16 | 2 | 0 | 0 | 0 | 0 | 1 | 1 |
|  | 1901-1950 | Exon16-Exon17 | 2 | 2 | 1 | 0 | 1 | 0 | 0 |
|  | 2001-2050 | Exon17-Exon18 | 2 | 1 | 1 | 0 | 0 | 0 | 1 |
| MSH2 | 1701-1750 | Exon11 | 19 | 2 | 2 | 0 | 0 | 1 | 16 |
|  | 1251-1300 | Exon7 | 6 | 1 | 1 | 0 | 0 | 4 | 1 |
|  | 1201-1250 | Exon7 | 4 | 2 | 1 | 1 |  | 1 | 1 |
|  | 301-350 | Exon2 | 3 | 2 | 1 | 0 | 1 | 0 | 1 |
|  | 1801-1850 | Exon12 | 3 | 2 | 2 | 0 | 0 | 1 | 0 |
|  | 2001-2050 | Exon12-Exon13 | 3 | 1 | 1 | 0 | 0 | 0 | 2 |
|  | 2051-2100 | Exon13 | 3 | 1 | 1 | 0 | 0 | 2 | 0 |
|  | 2601-2650 | Exon15 | 3 | 1 | 1 | 0 | 0 | 1 | 1 |
|  | 151-200 | Exon1 | 2 | 0 | 0 | 0 | 0 | 1 | 1 |
|  | 551-600 | Exon3 | 2 | 1 | 1 | 0 | 0 | 0 | 1 |
|  | 801-850 | Exon4-Exon5 | 2 | 1 | 0 | 0 | 1 | 0 | 1 |
|  | 1051-1100 | Exon6 | 2 | 0 | 0 | 0 | 0 | 1 | 1 |
|  | 1151-1200 | Exon7 | 2 | 1 | 0 | 0 | 1 | 0 | 1 |
|  | 1301-1350 | Exon7-Exon8 | 2 | 2 | 0 | 1 | 1 | 0 | 0 |
|  | 1601-1650 | Exon10 | 2 | 1 | 0 | 0 | 1 | 0 | 1 |
|  | 1901-1950 | Exon12 | 2 | 0 | 0 | 0 | 0 | 0 | 2 |
|  | 2151-2200 | Exon13 | 2 | 0 | 0 | 0 | 0 | 1 | 1 |
|  | 2201-2250 | Exon13-Exon14 | 2 | 2 | 0 | 0 | 2 | 0 | 0 |
|  | 2251-2300 | Exon14 | 2 | 0 | 0 | 0 | 0 | 1 | 1 |
| MSH6 | 3301-3350 | Exon5 | 6 | 3 | 2 | 1 | 0 | 2 | 1 |
|  | 3551-3600 | Exon6 | 6 | 0 | 0 | 0 | 0 | 0 | 6 |
|  | 3601-3650 | Exon6-Exon7 | 4 | 2 | 2 | 0 | 0 | 2 | 0 |
|  | 3251-3300 | Exon4-Exon5 | 3 | 2 | 0 | 0 | 2 | 1 | 0 |
|  | 1151-1200 | Exon4 | 2 | 2 | 0 | 0 | 2 | 0 | 0 |
|  | 2001-2050 | Exon4 | 2 | 2 | 0 | 0 | 2 | 0 | 0 |
|  | 3201-3250 | Exon4 | 2 | 2 | 1 | 0 | 1 | 0 | 0 |
|  | 3851-3900 | Exon8-Exon9 | 2 | 0 | 0 | 0 | 0 | 2 | 0 |
| PMS2 | 1751-1800 | Exon11 | 7 | 3 | 3 | 0 | 0 | 1 | 3 |
|  | 251-300 | Exon3-Exon4 | 2 | 1 | 1 | 0 | 0 | 0 | 1 |
|  | 851-900 | Exon8 | 2 | 2 | 2 | 0 | 0 | 0 | 0 |

**Supplemental table 3:**

(Summary of variants with multiple carriers and corresponding allele frequency data.)

| Gene | Exon/Intron | HGVSc | Clinical Significance | RS Number | Recurrence | Allele Frequency |  |  |  |  |
| --- | --- | --- | --- | --- | --- | --- | --- | --- | --- | --- |
|  |  |  |  |  |  | Project | Population | Sample Size | Ref Allele | Alt Allele |
| MLH1 | exon8 | c.649C>T | Uncertain | rs4986984 | 6 | 1000Genomes | East Asian | 1008 | C=0.9980 | T=0.0020 |
|  |  |  |  |  |  |  | South Asian | 978 | C=0.999 | T=0.001 |
|  |  |  |  |  |  | 3.5KJPNv2 | Japanese | 16760 | C=0.99678 | T=0.00322 |
|  |  |  |  |  |  | Allele Frequency Aggregator | Asian | 6288 | C=0.9959 | T=0.0041 |
|  |  |  |  |  |  | ExAC | Asian | 25156 | C=0.99845 | T=0.00155 |
|  |  |  |  |  |  | gnomAD - Exomes | Asian | 49010 | C=0.99812 | T=0.00188 |
|  |  |  |  |  |  | gnomAD - Genomes | East Asian | 3128 | C=0.9978 | T=0.0022 |
|  |  |  |  |  |  | Korean Genome Project | Korean | 1832 | C=0.9929 | T=0.0071 |
|  |  |  |  |  |  | KOREAN population from KRGDB | Korean | 2922 | C=0.9921 | T=0.0079 |
|  |  |  |  |  |  | The PAGE Study | Asian | 8318 | C=0.9953 | T=0.0047 |
| MLH1 | intron15 | c.1731+1G>A | Pathogenic | rs267607853 | 3 | No allele frequency data | | | | |
| PMS2 | exon11 | c.1738A>T | Pathogenic | rs267608169 | 3 | 3.5KJPNv2 | Japanese | 16758 | T=0.99994 | A=0.00006 |
| MLH1 | exon16 | c.1852_1854del | Pathogenic | None | 2 | No allele frequency data | | | | |
| MSH6 | exon5 | c.3205G>C | Uncertain | rs764113705 | 2 | 3.5KJPNv2 | JAPANESE | 16760 | G=0.99988 | C=0.00012 |
|  |  |  |  |  |  | Allele Frequency Aggregator | Asian | 112 | G=1.000 | C=0.000 |
|  |  |  |  |  |  |  | South Asian | 98 | G=1.00 | C=0.00 |
|  |  |  |  |  |  | ExAC | Asian | 25148 | G=0.99984 | C=0.00016 |
|  |  |  |  |  |  | gnomAD - Exomes | Asian | 48998 | G=0.99986 | C=0.00014 |
|  |  |  |  |  |  | gnomAD - Genomes | East Asian | 3132 | G=0.9990 | C=0.0010 |
| MLH1 | exon8 | c.676C>T | Pathogenic | rs63751615 | 2 | Allele Frequency Aggregator | Asian | 34 | C=1.00 | T=0.00 |
|  |  |  |  |  |  |  | South Asian | 6 | C=1.0 | T=0.0 |
|  |  |  |  |  |  | ExAC | Asian | 25118 | C=1.00000 | T=0.00000 |
|  |  |  |  |  |  | gnomAD - Exomes | Asian | 49002 | C=1.00000 | T=0.00000 |
|  |  |  |  |  |  | The PAGE Study | Asian | 8318 | C=1.0000 | T=0.0000 |
|  |  |  |  |  |  |  | SouthAsian | 856 | C=1.000 | T=0.000 |
| PMS2 | exon8 | c.861_864del | Pathogenic | rs267608154 | 2 | Allele Frequency Aggregator | Asian | 108 | (CTGT)3=1.000 | del(CTGT)2=0.000, delCTGT=0.000 |
|  |  |  |  |  |  |  | South Asian | 94 | (CTGT)3=1.00 | del(CTGT)2=0.00, delCTGT=0.00 |
|  |  |  |  |  |  | ExAC | Asian | 25100 | (CTGT)3=1.00000 | delCTGT=0.00000 |
|  |  |  |  |  |  | gnomAD - Exomes | Asian | 49008 | (CTGT)3=1.00000 | delCTGT=0.00000 |
| MSH2 | exon13 | c.2203A>G | Uncertain | rs2229061 | 2 | 1000Genomes | East Asian | 1008 | A=0.9990 | G=0.0010 |
|  |  |  |  |  |  | 3.5KJPNv2 | Japanese | 16760 | A=0.99952 | G=0.00048 |
|  |  |  |  |  |  | Allele Frequency Aggregator | Asian | 3132 | A=0.9987 | G=0.0013 |
|  |  |  |  |  |  | ExAC | Asian | 25162 | A=0.99992 | G=0.00008 |
|  |  |  |  |  |  | gnomAD - Exomes | Asian | 49010 | A=0.99996 | G=0.00004 |
|  |  |  |  |  |  | KOREAN population from KRGDB | Korean | 2922 | A=0.9993 | G=0.0007 |
|  |  |  |  |  |  | The PAGE Study | Asian | 8318 | A=0.9992 | G=0.0008 |
| MSH2 | intron5 | c.942+3A>T | Pathogenic | rs193922376 | 2 | Allele Frequency Aggregator | Asian | 108 | A=1.000 | T=0.000 |
|  |  |  |  |  |  | gnomAD - Exomes | Asian | 6182 | A=1.0000 | T=0.0000 |
|  |  |  |  |  |  | gnomAD - Genomes | East Asian | 8 | A=1.0 | T=0.0 |
| MSH6 | exon4 | c.1937A>G | Uncertain | rs201096652 | 2 | 1000Genomes | East Asian | 1008 | A=0.9990 | G=0.0010 |
|  |  |  |  |  |  | 3.5KJPNv2 | Japanese | 16760 | A=0.99952 | G=0.00048 |
|  |  |  |  |  |  | ExAC | Asian | 25160 | A=0.99976 | G=0.00024 |
|  |  |  |  |  |  | gnomAD - Exomes | Asian | 49010 | A=0.99982 | G=0.00018 |
| MLH1 | exon4 | c.350C>T | Pathogenic | rs63750781 | 2 | Allele Frequency Aggregator | Asian | 34 | C=1.00 | T=0.00 |
|  |  |  |  |  |  |  | South Asian | 6 | C=1.0 | T=0.0 |
|  |  |  |  |  |  | gnomAD - Exomes | Asian | 49006 | C=1.00000 | T=0.00000 |
|  |  |  |  |  |  | The PAGE Study | Asian | 8318 | C=1.0000 | T=0.0000 |
|  |  |  |  |  |  |  | SouthAsian | 856 | C=1.000 | T=0.000 |
| MSH6 | exon5 | c.3261del/dup | Pathogenic | rs267608078 | 2 | Allele Frequency Aggregator | Asian | 168 | (C)8=1.000 | delC=0.000, dupC=0.000, dupCC=0.000 |
|  |  |  |  |  |  |  | South Asian | 98 | (C)8=1.00 | delC=0.00, dupC=0.00, dupCC=0.00 |
